# Right Ventricular Diastolic Performance in Patients With Chronic Thromboembolic Pulmonary Hypertension Assessed by Echocardiography

**DOI:** 10.3389/fcvm.2021.755251

**Published:** 2021-11-25

**Authors:** Hong Meng, Wu Song, Sheng Liu, David Hsi, Lin-Yuan Wan, Hui Li, Shan-shan Zheng, Zhi-wei Wang, Rong Ren, Wei-xian Yang

**Affiliations:** ^1^Echocardiographic Imaging Center, National Center for Cardiovascular Diseases, Fuwai Hospital, Chinese Academy of Medical Sciences and Peking Union Medical College, Beijing, China; ^2^Department of Cardiac Surgery, National Center for Cardiovascular Diseases, Fuwai Hospital, Chinese Academy of Medical Sciences and Peking Union Medical College, Beijing, China; ^3^Heart and Vascular Institute, Stamford Hospital, Stamford, CT, United States; ^4^Department of Cardiac Surgery, Fuwai Hospital, Chinese Academy of Medical Sciences, Shenzhen, China; ^5^Department of Cardiology, Key Laboratory of Pulmonary Vascular Medicine, Fuwai Hospital, Chinese Academy of Medical Sciences, Beijing, China

**Keywords:** chronic thromboembolic pulmonary artery hypertension, right ventricular diastolic dysfunction, early diastolic strain rate, indexed right atrial area, right heart filling pressure

## Abstract

**Background:** There have been no systemic studies about right heart filling pressure and right ventricular (RV) distensibility in patients with chronic thromboembolic pulmonary hypertension (CTEPH). Therefore, we aimed to explore combinations of echocardiographic indices to assess the stages of RV diastolic dysfunction.

**Methods and Results:** We recruited 32 healthy volunteers and 71 patients with CTEPH. All participants underwent echocardiography, cardiac catheterization (in patients with CTEPH), and a 6-min walk test (6MWT). The right atrial (RA) end-systolic area was adjusted for body surface area (BSA) (indexed RA area). RV global longitudinal diastolic strain rates (SRs) and RV ejection fraction (EF) were measured by speckle tracking and three-dimensional echocardiography (3D echo), respectively. All 71 patients with CTEPH underwent pulmonary endarterectomy. Of the 71 patients, 52 (73%) had decreased RV systolic function; 12 (16.9%), 26 (36.6%), and 33 (46.5%) patients had normal RV diastolic pattern, abnormal relaxation (stage 1), and pseudo-normal patterns (stage 2), respectively. The receiver operating characteristic curve analysis showed that the optimal cut-off values of early diastolic SR <0.8 s^−1^ and indexed RA area > 8.8 cm^2^/BSA had the best accuracy in identifying patients with RV diastolic dysfunction, with 87% sensitivity and 82% specificity. During a mean follow-up of 25.2 months after pulmonary endarterectomy, the preoperative indexed RA area was shown as an independent risk factor of the decreased 6MWT distance.

**Conclusions:** Measuring early diastolic SR and indexed RA area would be useful in stratifying RV diastolic function.

## Introduction

Pulmonary arterial hypertension (PAH), left heart failure (HF), mechanical ventilation, and left ventricular assist device implantation are associated with right HF (1–3). Early recognition of right ventricular (RV) dysfunction may provide opportunities to optimize treatment strategy and delay the progression of heart failure. By the use of combined Doppler transtricuspid flow velocities and annular tissue Doppler velocity, RV diastolic dysfunction has been described (4). However, the methods were limited in some patients due to undetermined doppler patterns affected by heart rate, volume load, and other factors. To date, many studies have reported some hemodynamic indices of heart failure, including elevated central venous pressure, pulmonary capillary wedge pressure, increased left or RV pressure, and pulmonary artery pressure (PAP).

Recently, Mele et al. (5) reported 496 patients with HF and found patients with high left heart filling pressure had a worse prognosis than those with normal filling pressure and noticed that high mean RA pressure (>8 mmHg) could identify a subgroup of patients with worse prognosis. The relationship between right heart filling pressure, RV function, and outcomes in patients with PAH seem to be very important. As the right heart filling pressure increases, the RV systolic and/or diastolic dysfunction may deteriorate and influence clinical outcomes.

Our study aimed to explore echocardiographic parameters in assessing RV diastolic function, to prove the correlations between the right heart filling pressure and stages of RV diastolic function, and to observe the outcomes in patients with chronic thromboembolic pulmonary hypertension (CTEPH) after pulmonary endarterectomy.

## Materials and Methods

### Participants

The Institutional Review Board of Fuwai Hospital approved the study protocol (January 16, 2018, No. 2018-991), which was in accordance with the “Declaration of Helsinki.”

In total, 71 patients (mean age, 47.6 ± 13.6 years; 63% men) who were diagnosed with CTEPH at Fuwai Hospital between January 2018 and June 2020 were included. Their diagnosis was based on echocardiography, CT, and cardiac catheterization. None had a history of cancer. All these patients underwent pulmonary endarterectomy. Pre- and post-operative medical records of patients with CTEPH were collected.

Thirty-two healthy volunteers (mean age, 42.5 ± 8.0 years; 47% men) with no history of heart or lung diseases or symptoms and with normal physical examination findings were selected as control participants. Written informed consent was obtained from all participants.

### Echocardiography

Transthoracic echocardiography was performed using a 3–4.5-MHz transducer (Vivid E95, GE Vingmed Ultrasound, Horten, Norway). Measurements were performed according to the American Society of Echocardiography guidelines for the assessment of heart chambers (6). The right atrial (RA) end-systolic area was traced in the apical four-chamber view and adjusted for body surface area (BSA) (indexed RA area). RV end-diastolic and end-systolic areas were measured in the apical four-chamber view to calculate RV fractional area change (RV FAC). Tricuspid annular peak systolic excursion (TAPSE) was obtained using the M-mode echo of the lateral annulus.

Mitral and tricuspid inflow velocities were recorded. These parameters included early filling velocity (E), atrial filling velocity (A), E/A ratio, and deceleration time (DT). Mitral and tricuspid annular systolic velocity (s'), early-diastolic velocity (e'), and late-diastolic velocity (a') were assessed in the apical four-chamber view. The RV index of myocardial performance (RIMP) was defined as the ratio of the sum of the isovolumic contraction time and the isovolumic relaxation time (IVRT) divided by the ejection time.

**Figure 1:**
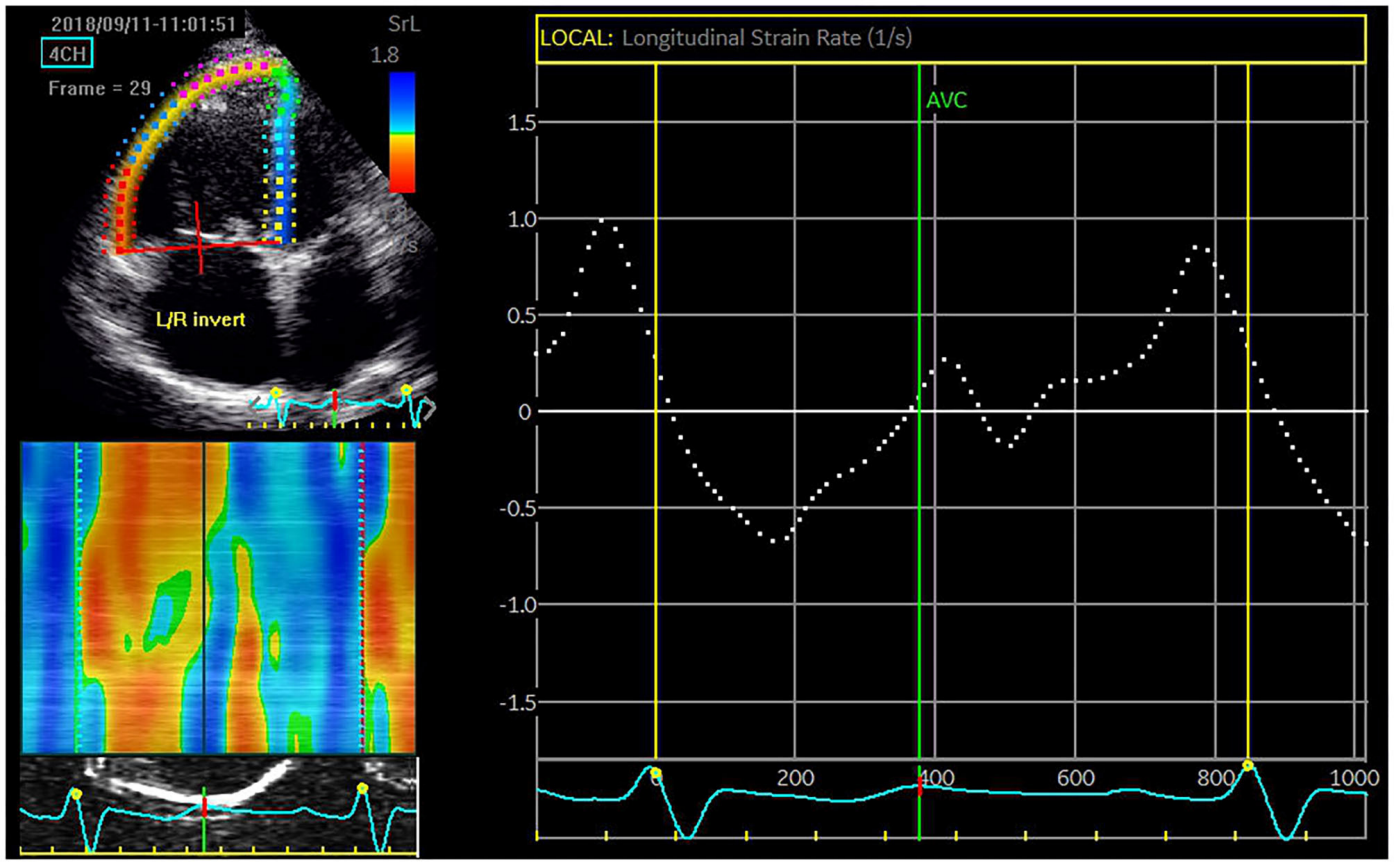
A six-segment model of the right ventricle was created by the tracking algorithm after manual delineation of the endocardial border. The global longitudinal strain rate (SR) (*dotted line*) is calculated by averaging the regional peak values. In this patient with chronic thromboembolic pulmonary artery hypertension (CTEPH), the global early diastolic SR is severely depressed (0.27 s^−1^).

Images were further analyzed using two-dimensional speckle echo software (EchoPAC, General Electric/Vingmed Ultrasound, Horten, Norway) with a calculation of RV global longitudinal strain (GLS), and early and late diastolic strain rates (SRs) for the entire traced contour of the right ventricle (Figure 1). Measurements were performed on an average of three beats.

Three-dimensional echocardiography (3D echo) was performed at the cardiac apex using a matrix-array transducer (4VC). All images were analyzed by four-dimensional RV analysis software (EchoPAC version 20, TomTec Imaging, Inc., Munich, Germany). RV end-diastolic volume (EDV) and RV end-systolic volume (ESV) were obtained. RV ejection fraction (EF) was then measured as the percentage change of the volumes.

Right ventricular systolic dysfunction was defined when at least two parameters were below the lower recommended limit of normal [i.e., RVEF ≤ 40%, TAPSE ≤ 17 mm, tricuspid valve (TV) annular s' ≤ 9.5 cm/s, FAC ≤ 35%, and/or GLS ≥ −20%] (7). We followed the criteria established by the American Society of Echocardiography for determining RV diastolic dysfunction in adults, grading it as normal (E/A ratio > 0.8), stage 1 (impaired relaxation: E/A <0.8), stage 2 (pseudo normal: E/A 0.8–2.1 with E/e′ >6), and stage 3 (restrictive filling: E/A > 2.1 with DT <120 ms) (4).

### Hemodynamic Measurements

In this study, all patients underwent left- and right-sided cardiac catheterization before or after echocardiography within 3 days. RA pressure, RV pressure, and PAP were obtained during systole and diastole. Left-heart catheterization was performed to exclude significant coronary artery disease or left-sided valve disease and to obtain LV pressure. Cardiac output was calculated and was adjusted by BSA as a cardiac index. Pulmonary vascular resistance was subsequently calculated and expressed as dynes.s.cm^−5^.

### Statistical Analysis

SPSS 24.0 was used for statistical analysis. Results were expressed as mean (±standSD) unless stated otherwise. When comparison among three groups was needed, a one-way ANOVA was carried out. The independent samples *t*-test was chosen to analyze differences between the two groups. The correlation was analyzed by performing a bivariate linear regression estimation. The diagnostic accuracy was determined by receiver operating characteristic (ROC) curve analysis. The chi-square test was used to compare proportions. Differences were considered significant if the *p* < 0.05.

## Results

### CTEPH Groups vs. Normal Control Participants

The baseline characteristics of all control participants and medical records of patients with CTEPH are shown in Table 1. In this study, 44 of 71 (62%) patients presented with New York Heart Association (NYHA) function class III or IV heart failure. Forty-nine (69%) patients completed the 6MWT. Among the 71 patients with CTEPH, 22 patients had trace tricuspid regurgitation (TR), 27 had mild TR, 17 had moderate TR, and five had severe TR. Fifty-two patients had RV EF ≤ 40%, 41 had TAPSE ≤ 17 mm, 26 had TV annular s' ≤ 9.5 cm/s, 42 had RV FAC ≤ 35%, and 52 had GLS < -20%. In total, 52 patients presented with decreased RV systolic function. Normal RV diastolic pattern, abnormal relaxation (stage 1), and pseudonormal patterns (stage 2) were present in 12 (16.9%), 26 (36.6%), and 33 (46.5%) of 71 patients, respectively, based on the TV E/A ratio, E/e' ratio, and DT.

**Table 1:** Mean values and SDs of the baseline characteristics of all participants and preoperative general medical records of CTEPH patients.

**Variables**		**Control** **(*n* = 32)**	**CTEPH patients** **(*n* = 71)**	***P*-Value**
Age, years		42.5 ± 8.0	47.6 ± 13.6	0.052
Male, no. (%)		15 (46.9%)	45 (63.4%)	0.134
SBP/DPB, mmHg		121/72	114/70	0.329
BMI		23.7 ± 2.4	23.3 ± 3.4	0.608
BSA		1.8 ± 0.2	1.8 ± 0.2	0.234
Blood glucose		5.0 ± 0.2	4.5 ± 0.6	
Hemoglobin (g/dL)		130 ± 5.6	140 ± 20	
**Renal function**				
BUN (mmol/L)		4.5 ± 0.4	5.7 ± 1.6	
Creatinine (mol/L)		61.8 ± 10.2	85.2 ± 16.0	
**Liver function**				
Total bilirubin (mol/L)		7.5 ± 2.5	17.2 ± 9.4	
Direct bilirubin (mol/L)		1.4 ± 0.3	4.7 ± 3.9	
NT pro-BNP			799.0 ± 882.1 (5 ~ 4,585)	
NYHA	I	32 (100%)	0 (0%)	
	II		27 (38%)	
	III		42 (59.2%)	
	IV		2 (2.8%)	
6MWT	≤ 300 m		12 (24.5%)	
	300–375 m		11 (22.4%)	
	375–449.9 m		17 (34.7%)	
	≥450 m	32 (100%)	9 (18.4%)	
GFR	≥90 ml/min	32 (100%)	34 (47.9%)	
	60–89 ml/min		25 (35.2%)	
	30–59 ml/min		10 (14.1%)	
	15–29 ml/min		2 (2.8%)	

*CTEPH, chronic thromboembolic pulmonary hypertension; BMI, body mass index; BSA, body surface area; BUN, blood urea nitrogen; NT pro-BNP, N-terminal prohormone of brain natriuretic peptide; 6MWT, 6-minute walk test; GFR, glomerular filtration rate*.

### Correlation Between Echocardiographic Indices and Right Heart Filling Pressure

**Figure 2:**
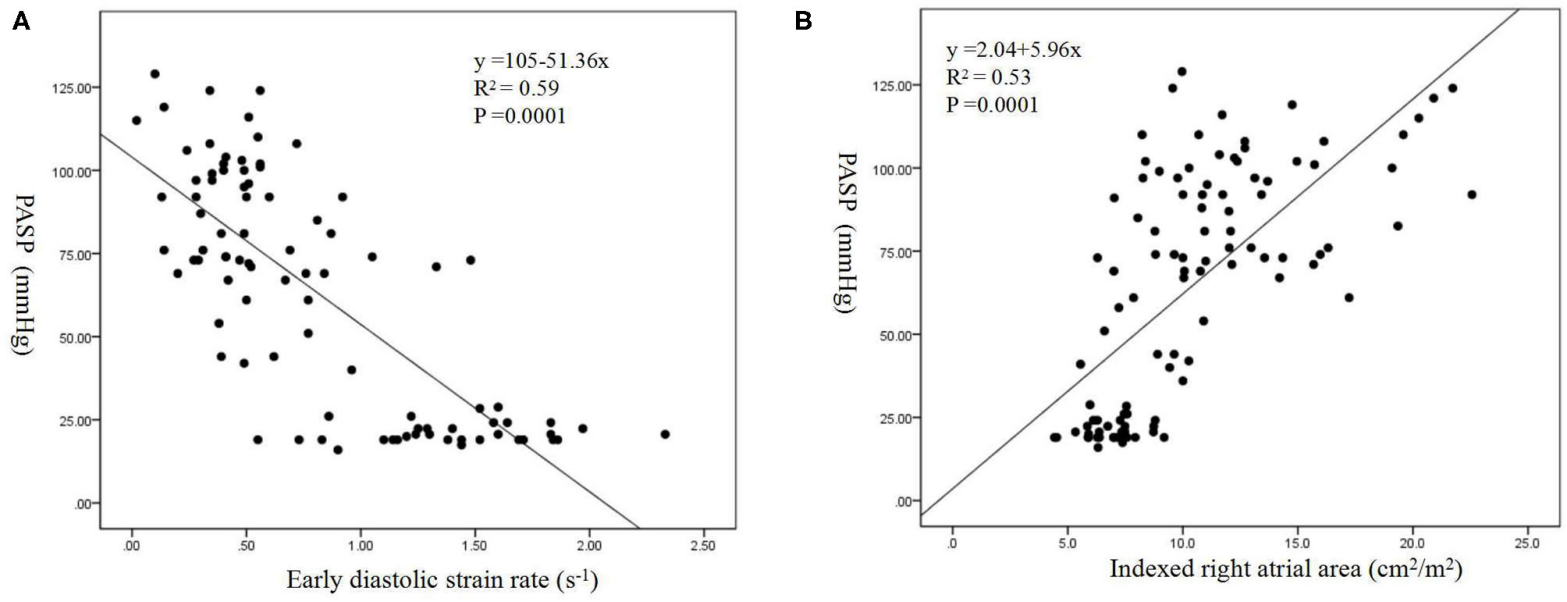
Correlations between pulmonary artery systolic pressure (PASP) and early diastolic SR **(A)** and indexed right atrial (RA) area **(B)**.

Moderate correlations were found between PASP, mean PAP and RIMP (*R* = 0.67 and 0.68, both *p* = 0.0001), IVRT (*R* = 0.60 and 0.60, both *p* = 0.0001), TV annular e' (*R* = −0.62 and −0.61, both *p* = 0.0001), early diastolic SR (*R* = −0.77 and −0.71, both *p* = 0.0001), and indexed RA area (*R* = 0.73 and 0.72, both *p* = 0.0001). The linear regression model showed that the indexed RA area was mostly impacted by increased PASP (β = 2.84, *p* = 0.0001) and mean PAP (β = 1.4, *p* = 0.001), including RIMP, IVRT, TV e', early diastolic SR, indexed RA area, TV E/A ratio, TV E/e' ratio, and TV DT (Figure 2).

**Figure 3:**
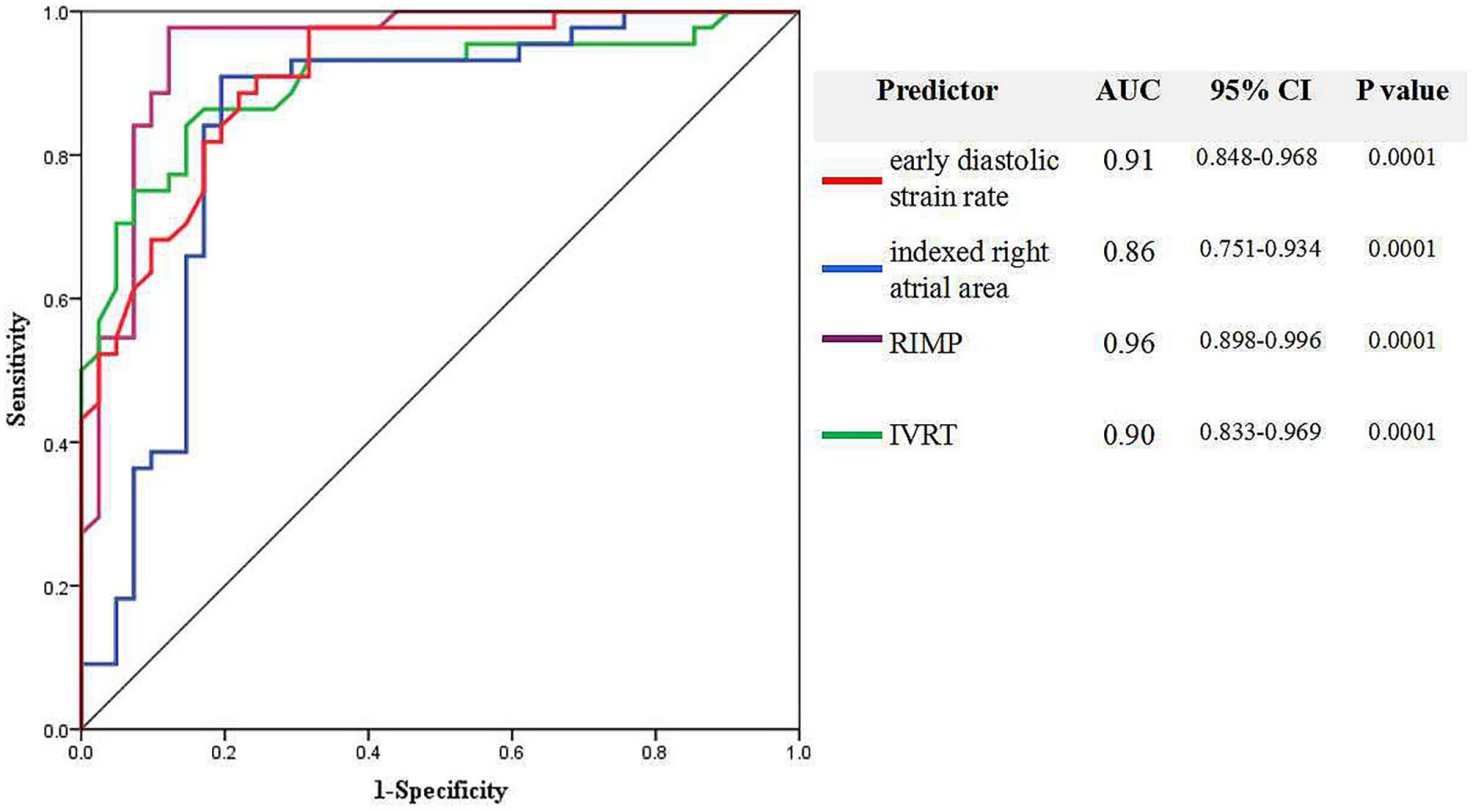
Receiver operating characteristic (ROC) curve analysis finds the echocardiographic parameters to identify right ventricular diastolic dysfunction based on early diastolic SR, indexed RA area, right ventricular index of myocardial performance (RIMP), and isovolumic relaxation time (IVRT).

Receiver operating characteristic curve analysis showed that the optimal cut-off values of early diastolic SR <0.8 s^−1^, RIMP >0.45, IVRT >82 ms, and indexed RA area >8.8 cm^2^/m^2^ had the best accuracy in identifying RV diastolic dysfunction, with sensitivities of 82, 97, 84, and 87; specificities of 84, 87, 87, and 82%; and areas under the curve of 0.91, 0.96, 0.90, and 0.86, respectively (Figure 3). RV diastolic dysfunction was detected in 67 of 71 (94.4%) patients with CTEPH, when at least two novel parameters were beyond the recommended limit of normal (i.e., early diastolic SR <0.8 s^−1^, RIMP >0.45, IVRT >82 ms, indexed RA area >8.8 cm^2^/m^2^). Furthermore, early diastolic SR <0.31 s^−1^ and indexed RA area >10 cm^2^/m^2^ could further separate stage 2 RV diastolic dysfunction from stage 1, with sensitivities of 74% and 74%, and specificities of 27 and 53%, respectively.

Table 2 summarizes echocardiographic parameters assessing RV systolic and diastolic function in normal control participants and in patients with CTEPH with the normal or elevated right heart filling pressures. The patients with CTEPH with mean RA pressure ≤ 8 mmHg or RV diastolic pressure ≤ 4 mmHg had better diastolic function indices than the patients with CTEPH with mean RA pressure >8 mmHg or RV diastolic pressure >4 mmHg (8, 9). With the elevation of mean RA pressure or RV diastolic pressure, the proportions of the patients with RV diastolic dysfunction increased, especially those with stage II, with chi-square values of 4.89 (*p* = 0.08) and 11.86 (*p* = 0.003). When early diastolic SR, indexed RA area, RIMP, and IVRT were added, the detection rate of RV diastolic dysfunction was increased, with chi-square values of 6.71 (*p* = 0.03) and 5.36 (*p* = 0.04).

**Table 2:** Right ventricular systolic and diastolic parameters of normal control participants and CTEPH patients with different right atrial and right ventricular pressures.

**Variables**		**Control**	**CTEPH patients**		**CTEPH patients**	
			**With RAMP ≤8 mmHg**	**With >8 mmHg**	**With RVDP ≤4 mmHg**	**With >4 mmHg**
		**(Group 1)**	**(Group 2)**	**(Group 3)**	**(Group 4)**	**(Group 5)**
Numbers		32	52	19	46	25
PASP (mm Hg, by echo)		21.09 ± 3.09	76.06 ± 26.75^a^	91.80 ± 22.15^a,j^	73.98 ± 25.66^a^	92.11 ± 24.60^a,h^
RV EDV (ml, by 3D echo)		72.07 ± 18.16	129.67 ± 54.63^a^	157.86 ± 56.58^a^	136.63 ± 59.28^a^	140.31 ± 52.45^a^
RV EF (%, by 3D echo)		56.27 ± 3.13	33.89 ± 10.39^a^	29.90 ± 9.68^a^	33.65 ± 10.68^a^	31.27 ± 9.77^a^
RIMP		0.28 ± 0.09	0.71 ± 0.32^a^	0.63 ± 0.17^a^	0.66 ± 0.27^a^	0.75 ± 0.32^a^
TVE/A ratio		1.36 ± 0.25	0.97 ± 0.32^a^	1.26 ± 0.46^j^	0.95 ± 0.33^a^	1.21 ± 0.41^i^
TV annular e' (cm/s)		11.58 ± 2.47	7.26 ± 1.89^a^	7.93 ± 3.01^a^	7.51 ± 2.09^a^	7.44 ± 2.57^a^
E/e' ratio		4.94 ± 1.19	6.44 ± 2.61^c^	7.74 ± 2.63^b^	6.44 ± 2.88^c^	7.59 ± 1.98^a^
TV DT (ms)		262.09 ± 73.83	240.20 ± 92.32	220.07 ± 67.98	245.11 ± 89.86	210.94 ± 75.17^d^
IVRT (ms)		57.50 ± 18.46	122.88 ± 54.17^a^	97.66 ± 32.71^a,j^	112.93 ± 50.53^a^	119.29 ± 50.70^a^
Indexed RA area (cm^2^/m^2^)		6.82 ± 1.10	11.36 ± 3.47^a^	14.24 ± 4.73^a,j^	11.67 ± 3.82^a^	12.91 ± 4.33^a^
Early diastolic SR (s^−1^)		1.40 ± 0.39	0.51 ± 0.29^a^	0.65 ± 0.34^a^	0.52 ± 0.29^a^	0.57 ± 0.33^a^
Late diastolic SR (s^−1^)		1.14 ± 0.32	0.94 ± 0.29^c^	0.59 ± 0.32^a,f^	0.84 ± 0.28^a^	0.87 ± 0.40^d^
Mean RA pressure (mm Hg)			4.37 ± 2.34	12.00 ± 3.27^e^	5.76 ± 3.9	7.61 ± 4.8^a^
RV diastolic pressure (mm Hg)			1.20 ± 5.95	3.83 ± 4.06	−0.53 ± 5.12	6.91 ± 2.14^g^
RV diastolic pattern1	Normal	32	8	4	10	2
	Stage 1		23	3	22	4
	Stage 2		21	12(χ^2^ = 4.89)	15	18 (χ^2^ = 11.86)^h^
RV diastolic pattern2	Normal	32	2	2	3	1
	Stage 1		22	2	20	4
	Stage 2		28	15 (χ^2^ = 6.71)	24	(χ^2^ =5.36)^i^

*CTEPH, chronic thromboembolic pulmonary artery hypertension; RAMP, right atrial mean pressure; RVDP, right ventricular diastolic pressure; PASP, pulmonary artery systolic pressure; 3D echo, three-dimensional echocardiography; RV EDV, right ventricular end-diastolic volume; EF, ejection fraction; RIMP, right ventricular index of myocardial performance; DT, deceleration time; IVRT, isovolumic relaxation time; RA area, right atrial area; SR, strain rate*.

*RV diastolic pattern1, based on the E/A ratio, E/e′ ratio, and DT*.

*RV diastolic pattern2, added early diastolic strain rate, indexed right atrial area, RIMP, and IVRT*.

*In comparison with group 1*,

a *P = 0.0001*;

b *P = 0.001*;

c *P < 0.007*;

d *P < 0.03*.

*In comparison with group 2*,

e *P = 0.0001*;

f *P = 0.001*;

j *P ≤ 0.03*.

*In comparison with group 4*,

g *P = 0.0001*;

h *P < 0.01*;

i *P ≤ 0.04*.

### Follow-Up

We followed 68 patients with CTEPH from 1 to 45 (25.2 ± 9.3) months after pulmonary endarterectomy. Three patients were lost for follow-up after discharge. During the follow-up period, three late deaths occurred (4.4%). One patient died from advanced gastric cancer with gastrointestinal hemorrhage, and two patients died from a lung infection, probably related to residual pulmonary hypertension. Furthermore, 61 of 65 patients (93.8%) were in NYHA I or II. Among the 65 patients, 57 (87.7%) patients had mild or trace TR, five (7.7%) patients had mild-to-moderate TR, and the remaining three (4.6%) patients had moderate TR. Finally, 34 of 63 (54%) patients completed the 6MWT during follow-up. Compared with the 25 patients with a 6MWT distance ≥450 m, the nine patients whose 6MWT distance ranged from 375 to 499.9 m had a larger preoperative indexed RA area (14.2 ± 3.8 vs 10.1 ± 2.5, *p* = 0.002). Univariate logistic regression analysis showed that only preoperative indexed RA area was significantly associated with the decreased 6MWT distance during follow-up among all related factors, including preoperative mean PAP, PASP, TR degree, early diastolic SR, RIMP, IVRT, the follow-up PASP, and TR degree (*p* = 0.023). Furthermore, the multivariate logistic regression model found that the preoperative indexed RA area was an independent risk factor of the decreased 6MWT distance during follow-up (Table 3).

**Table 3:** The relations between the relative factors and the decreased 6MWT distance during follow-up based on logistic regression models, including preoperative pulmonary artery systolic pressure (PASP), mean pulmonary artery pressure (PAP), indexed right atrial area, early diastolic strain rate, right ventricular index of myocardial performance, isovolumic relaxation time, tricuspid regurgitation (TR) degree, the follow-up PASP, and TR degree.

**Variables**	**Univariate logistic regression model**				**Multivariate logistic regression model**			
	**B**	**Ward**	**Exp (B)**	***P*-Value**	**B**	**Ward**	**Exp (B)**	***P*-Value**
PASP (mm Hg)	0.004	0.04	1.004	0.841	−0.080	0.757	0.923	0.384
PAP (mean, mm Hg)	0.024	0.50	1.024	0.479	0.173	1.225	1.189	0.268
Indexed RA area (cm^2^/m^2^)	0.319	5.193	1.375	0.023	3.383	3.949	1.606	0.036
Early diastolic SR (s^−1^)	0.649	0.266	1.913	0.606	1.830	0.946	6.235	0.331
RIMP	−1.156	0.474	0.315	0.491	−3.034	0.417	0.048	0.519
IVRT (ms)	−0.219	0.456	0.993	0.50	0.004	0.026	1.004	0.872
TR degree	0.802	0.721	2.057	0.37	−0.932	0.382	0.394	0.537
TR degree (FU)	0.975	−0.997	0.369	0.324	3.891	1.105	0.323	0.293
PASP (FU, mm Hg)	0.003	0.003	1.003	0.957	−0.223	1.853	0.800	0.173

*PASP, pulmonary artery systolic pressure; PAP (mean), pulmonary artery mean pressure; RA area, right atrial area; SR, strain rate; RIMP, right ventricular index of myocardial performance; IVRT, isovolumic relaxation time; TR, tricuspid regurgitation; FU, follow up*.

## Discussion

This study analyzed the echocardiographic parameters assessing RV diastolic performance and hemodynamic data measured by right-heart catheterization. The main findings of this study are as follows: (1) early diastolic SR and indexed RA area were useful in stratifying RV diastolic dysfunction; (2) stages of RV diastolic dysfunction were related to the degree of the increased right heart filling pressure, and (3) preoperative indexed RA area could predict postoperative 6MWT distance.

The criteria recommended by the American Society of Echocardiography for RV diastolic function evaluation have been described, with differences in the E/A ratio, E/e' ratio, and DT. However, some patients had overlapping E- and A-waves and were markedly influenced by the respiratory cycle, especially the patients with PAH or CTEPH. Consequently, this would hamper the assessment of the appropriate and reproducible RV diastolic function.

### Additional Indices to Stratify RV Diastolic Function

In an earlier report from our laboratory, we found good correlations between PASP and echocardiographic parameters assessing RV diastolic function (10). Our evaluation further confirmed that PASP was significantly related to early diastolic SR and indexed RA area.

Our study found that RV-impaired relaxation pattern (stage 1) mostly occurred in patients with CTEPH with normal right heart filling pressure. The proportion of patients with stage 2 RV diastolic dysfunction was higher among those with the increased right heart filling pressure. RV diastolic dysfunction was an early sign of myocardial remodeling in CTEPH, and it presented earlier than the actual increase in RA or ventricular filling pressure (11).

In the chronic setting, as the ventricle thickens, RV filling becomes more dependent on RA performance driven by progressive perturbation of the normal Frank-Starling mechanism to preserve cardiac output. With RV deteriorating diastolic function, increased RA contractility and right heart filling pressure caused the RA chamber to distend. Maniar et al. (12) reported a 33% increase in RA elastance and a 45% increase in RA-diastolic stiffness during acute RV pressure overload. Nagueh et al. (13) confirmed that RA volume was significantly related to mean RA pressure, and the specificity of minimal RA volume (>30 mm^3^) for separating normal from elevated mean RAP (>8 mmHg) was approximately 90%. A Canadian study (14) showed that increased indexed RA volume (>33 ml/m^2^) and RA area (>20 cm^2^) could detect elevated RV end-diastolic pressure with a sensitivity of 90% and a specificity of 80%. Our study confirmed that indexed RA area >8.8 and >10 cm^2^/m^2^ were helpful to identify stage 1 and stage 2 RV diastolic dysfunction. Unlike the findings of previous studies, we measured the indexed RA area, instead of the indexed RA volume, since the accuracy of RA volume was limited. Until now, the three-dimensional echo software, which is used to measure RA volume, is developed for LV analysis.

The early rapid and active filling depends more on the ventricular myocardial relaxation which could be assessed by SR accurately (15–17). Okumura et al. (18) found that early diastolic SR is significantly correlated with the time constant of RV relaxation measured by cardiac catheterization. Early diastolic SR was an independent predictor of RV relaxation time in children with PAH without intracardiac shunts.

Thus, the indexed RA area and early diastolic SR could be used to evaluate the stages of RV diastolic function, especially when the E/A and E/e' ratios could not be accurately obtained. To enhance the accuracy and reliability of RV diastolic dysfunction assessments, the new diagnostic model was tested based on at least two abnormal results of the four novel parameters in this study, including indexed RA area, early diastolic SR, RIMP, and IVRT.

### Predictive Value of the Indexed RA Area

Recent data suggested that decreased 6MWT was associated with lower LV EF, LV diastolic dysfunction, and increased left atrial dimensions (19, 20). Some researchers found that RA size and function correlated well with the functional capacity of the patients with known right heart involvement, such as in idiopathic PAH or chronic obstructive pulmonary disease (21, 22). Nógrádi et al. investigated 80 patients with systemic sclerosis and reported that RA stiffness (ratio of TV E/e' to RA reservoir strain) was an independent predictor of 6MWT distance (21). Faludi et al. (22) reported that RV diastolic function and RV filling pressure significantly correlated with 6MWT distance, and the indexed RA area was an independent predictor of 6MWT distance in patients with chronic obstructive pulmonary disease. Our study found that markedly increased preoperative indexed RA area could predict decreased exercise performance of patients with CTEPH after pulmonary endarterectomy. One possible explanation for this phenomenon is that the larger RA size and higher right heart filling pressure may impair atrial function more seriously and require longer recovery periods or indicate irreversible impairment. The RA size could reflect ventricular relaxation state and predict exercise performance.

### Study Limitations

This is a single-center study, albeit prospective, with some limitations. RV diastolic function is determined by multiple factors at the cellular, myocardial, and heart chamber size levels. Right heart filling pattern and filling pressures reflect the net balance of many variables. It is difficult to capture all these variables by either invasive or non-invasive techniques. We feel that RV diastolic dysfunction could not be adequately assessed by a single parameter. Some investigators suggested that a diastolic RV pressure increase by >4 mmHg or end-diastolic pressure >10 mmHg could define RV diastolic dysfunction, but we did not measure pressure waveforms generated by the pulmonary artery catheters or monitor pressure-volume loops by conductance catheters in this study.

## Conclusions

We found that RV diastolic dysfunction is a common feature in patients with CTEPH. Measurements of early diastolic SR and indexed RA area were very helpful in stratifying RV diastolic dysfunction. RV diastolic dysfunction preceded RV systolic dysfunction and might occur earlier before the RA or ventricular filling pressure increase. An increased preoperative indexed RA area might predict a decreased postoperative exercise performance.

## Data Availability Statement

The raw data supporting the conclusions of this article will be made available by the authors, without undue reservation.

## Ethics Statement

The Institutional Review Board of Fuwai Hospital approved the study protocol (January 16, 2018, No. 2018-991), and the procedures followed were in accordance with the Declaration of Helsinki. The patients/participants provided their written informed consent to participate in this study.

## Author Contributions

HM collect and analyze data and draft manuscript. WS collect data and follow up the patients. SL perform pulmonary endarterectomy. DH discuss the results and revise this manuscript. L-YW and HL collect echo data. S-sZ, Z-wW, and RR collect clinical data. W-xY collect the patients and treat them at clinics. All authors contributed to the article and approved the submitted version.

## Funding

This work was supported by the Chinese Academy of Medical Sciences Innovation Fund for Medical Sciences (2017-I2M-3-003) and Capital Clinical feature application research and achievements promotion (Z-171100001017215).

## Conflict of Interest

The authors declare that the research was conducted in the absence of any commercial or financial relationships that could be construed as a potential conflict of interest.

## Publisher's Note

All claims expressed in this article are solely those of the authors and do not necessarily represent those of their affiliated organizations, or those of the publisher, the editors and the reviewers. Any product that may be evaluated in this article, or claim that may be made by its manufacturer, is not guaranteed or endorsed by the publisher.

## References

[1] Yu CM Sanderson JE Chan S Yeung L Hung YT Woo KS. Right ventricular diastolic dysfunction in heart failure. Circulation. (1996) 93:1509–14. 10.1161/01.CIR.93.8.1509 8608618

[2] Hiemstra YL Debonnaire P Bootsma M Schalij MJ Bax JJ Delgado V. Prevalence and prognostic implications of right ventricular dysfunction in patients with hypertrophic cardiomyopathy. Am J Cardiol. (2019) 124:604–12. 10.1016/j.amjcard.2019.05.021 31204037

[3] Zhu L Yang YH Wu YF Zhai ZG Wang C. Value of right ventricular dysfunction for prognosis in pulmonary embolism. Int J Cardiol. (2008) 127:40–5. 10.1016/j.ijcard.2007.06.093 17716753

[4] Rudski LG Lai WW Afilalo J Hua LQ Handschumacher MD Chandrasekaran K. Guidelines for the echocardiographic assessment of the right heart in adults: a report from the American society of echocardiography endorsed by the European association of echocardiography, a registered branch of the European society of cardiology, and the Canadian society of echocardiography. J Am Soc Echocardiogr. (2010) 23:685–713. 10.1016/j.echo.2010.05.010 20620859

[5] Mele D Pestelli G Molin DD Smarrazzo V Andrea GL Trevisan F. Right atrial pressure is associated with outcomes in patients with heart failure and indeterminate left ventricular filling pressure. J Am Soc Echocardiogr. (2020) 33:1345–56. 10.1016/j.echo.2020.05.027 32741596

[6] Lang RM Badano LP Mor-Avi V Afilalo J Armstrong A Ernande L. Recommendations for cardiac chamber quantification by echocardiography in adults: an update from the American society of echocardiography and the European association of cardiovascular imaging. J Am Soc Echocardiogr. (2015) 28:1–39.e14. 10.1016/j.echo.2014.10.003 25559473

[7] Amsallem MD Kuznetsova T Hanneman K Denault A Haddad F. Right heart imaging in patients with heart failure: a tale of two ventricles. Curr Opin Cardiol. (2016) 31:469–82. 10.1097/HCO.0000000000000315 27467173 PMC5133417

[8] Hoette S Creuzé N Günther S Montani D Savale L Jaïs X. RV fractional area change and tapse as predictors of severe right ventricular dysfunction in pulmonary hypertension: a CMR study. Lung. (2018) 196:157–164. 10.1007/s00408-018-0089-7 29435740

[9] Krishnan A Markham R Savage M Wong YW Walters D. Right heart catheterisation: how to do it. Heart Lung Circul. (2018) 1:1–8. 10.1016/j.hlc.2018.08.005 30253970

[10] Meng H Chandrasekaran K Villarraga HR Shah AA Kittipovanonth M Cha SS. Right and left ventricular interaction in pulmonary hypertension: insight from velocity vector imaging. Echocardiography. (2019) 36:877–87. 10.1111/echo.14328 30985965

[11] DiLorenzo M Hwang WT Goldmuntz E Ky B Rosa LM. Diastolic dysfunction in tetralogy of fallot: comparison of echocardiography with catheterization. Echocardiography. (2018) 35:1641–8. 10.1111/echo.14113 30105757 PMC6205242

[12] Maniar HS Prasad SM Gaynor SL Chu CM Steendijk P Moon MR. Impact of pericardial restraint on right atrial mechanics during acute right ventricular pressure load. Am J Physiol Heart Circ Physiol. (2003) 284:H350–7. 10.1152/ajpheart.00444.2002 12388317

[13] Nagueh SF Kopelen HA Zoghbi WA. Relation of mean right atrial pressure to echocardiographic and doppler parameters of right atrial and right ventricular function. Circulation. (1996) 93:1160–9. 10.1161/01.CIR.93.6.1160 8653837

[14] Addetia K Sebag IA Marelli A Do DH Afilalo J Martucci G. Right ventricular end-diastolic wall stress: does it impact on right atrial size, and does it differ in right ventricular pressure vs volume loading conditions? Can J Cardiol. (2013) 29:858–65. 10.1016/j.cjca.2012.06.024 23062663

[15] Puwanant S Park M Popović ZB Tang WH Farha S George D. Ventricular geometry, strain, and rotational mechanics in pulmonary hypertension. Circulation. (2010) 121:259–66. 10.1161/CIRCULATIONAHA.108.844340 20048214 PMC2846516

[16] Fukuda Y Tanaka H Sugiyama D Ryo K Onishi T Fukuya H. Utility of right ventricular free wall speckle-tracking strain for evaluation of right ventricular performance in patients with pulmonary hypertension. J Am Soc Echocardiogr. (2011) 24:1101–8. 10.1016/j.echo.2011.06.005 21775102

[17] Herod JW Ambardekar AV. Right ventricular systolic and diastolic function as assessed by speckle-tracking echocardiography improve with prolonged isolated left ventricualr assist device support. Clin Invest. (2014) 20:498–505. 10.1016/j.cardfail.2014.04.017 24785158

[18] Okumura K Slorach C Mroczek D Dragulescu A Mertens L Redington AN. Right ventricular diastolic performance in children with pulmonary arterial hypertension associated with congenital heart disease correlation of echocardiographic parameters with invasive reference standards by high-fidelity micromanometer catheter. Circ Cardiovasc Imaging. (2014) 7:491–501. 10.1161/CIRCIMAGING.113.001071 24577356

[19] Gardin JM Leifer ES Fleg JL Whellan D Kokkinos P LeBlanc MH. Relationship of doppler-echocardiographic left ventricular diastolic function to exercise performance in systolic heart failure: the HF-ACTION study. Am Heart J. (2009) 158:S45–52. 10.1016/j.ahj.2009.07.015 19782788 PMC2950162

[20] Bajraktari G Dini FL Fontanive P Elezi S Berisha V Napoli AM. Independent and incremental prognostic value of doppler-derived left ventricular total isovolumic time in patients with systolic heart failure. Int J Cardiol. (2011) 148:271–5. 10.1016/j.ijcard.2009.09.567 19948365

[21] Nógrádi A Porpáczy A Porcsa L Minier T Czirják L Komócsi A. Relation of right atrial mechanics to functional capacity in patients with systemic sclerosis. Am J Cardiol. (2018) 122:1249–54. 10.1016/j.amjcard.2018.06.021 30082039

[22] Faludi R Hajdu M Vértes V Nógrádi A Varga N Illés MB. Diastolic dysfunction is a contributing factor to exercise intolerance in COPD. COPD. (2016) 13:345–51. 10.3109/15412555.2015.1084614 26682932

