# Supplementary figures and images for: Right Ventricular Diastolic Performance in Patients With Chronic Thromboembolic Pulmonary Hypertension Assessed by Echocardiography
Source: Front Cardiovasc Med. 2021 Nov 25;8:755251. doi: 10.3389/fcvm.2021.755251 (PMC8660143; doi:10.3389/fcvm.2021.755251)

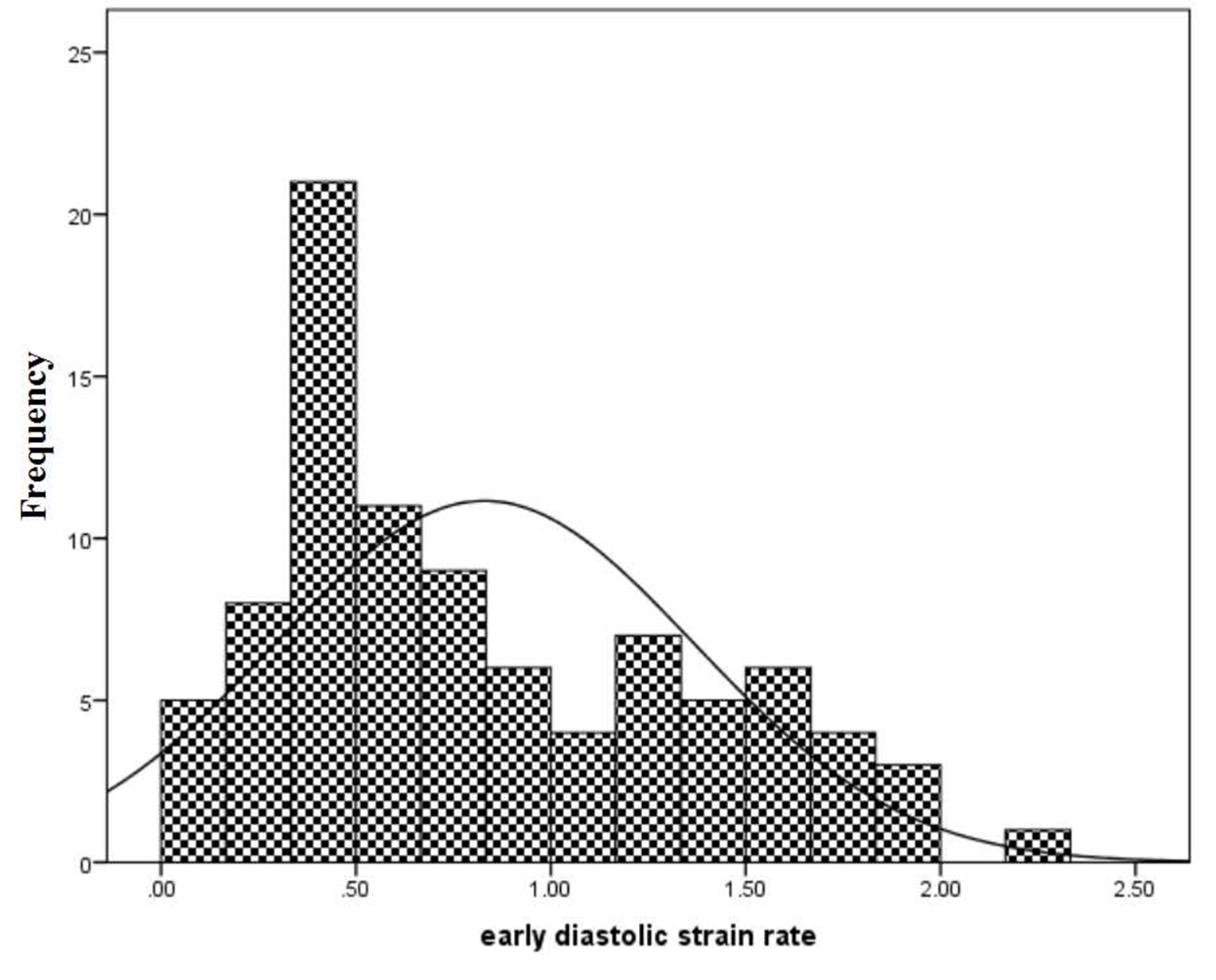

Supplement: Supplementary file 1 [file Image_1.JPEG]

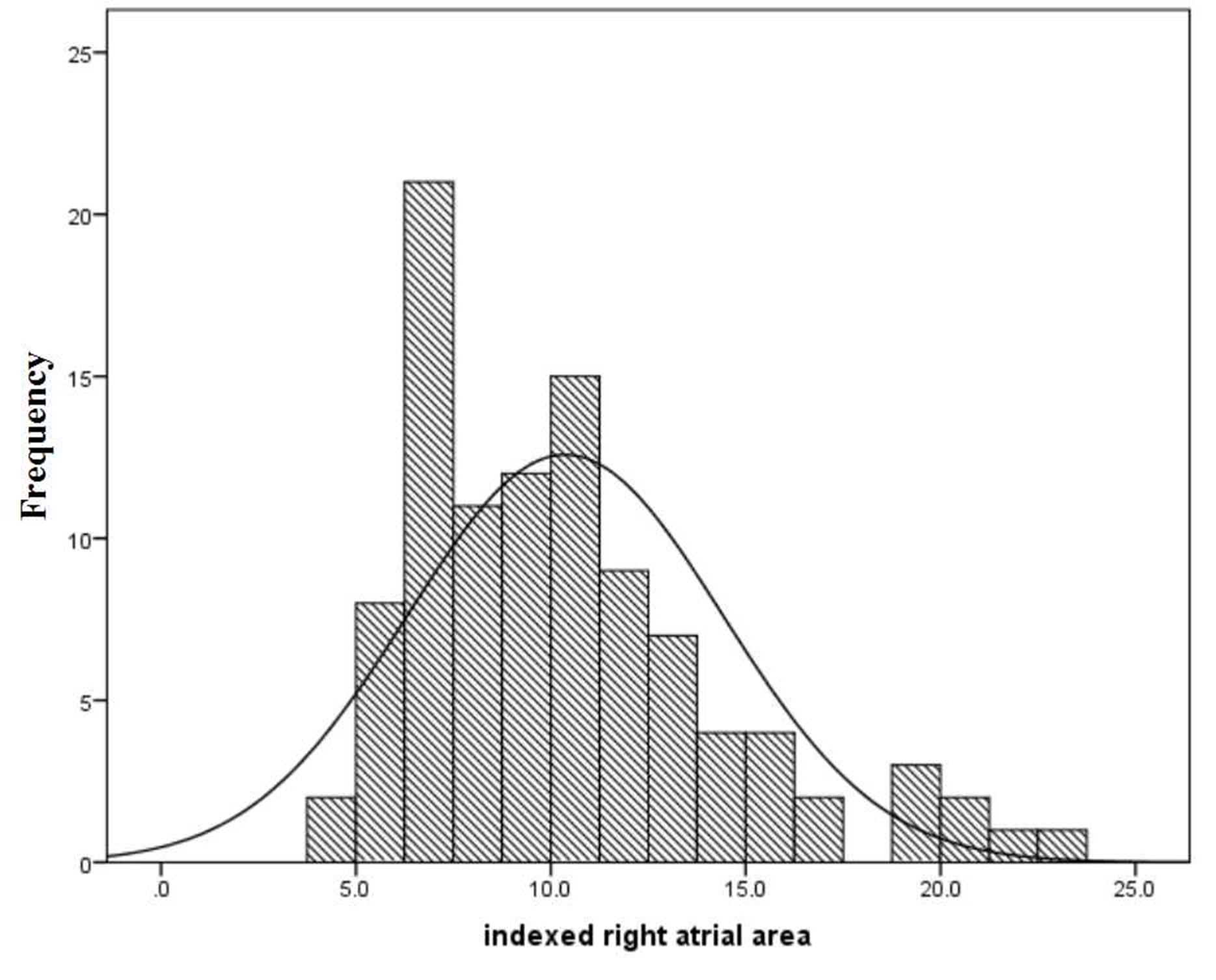

Supplement: Supplementary file 2 [file Image_2.JPEG]

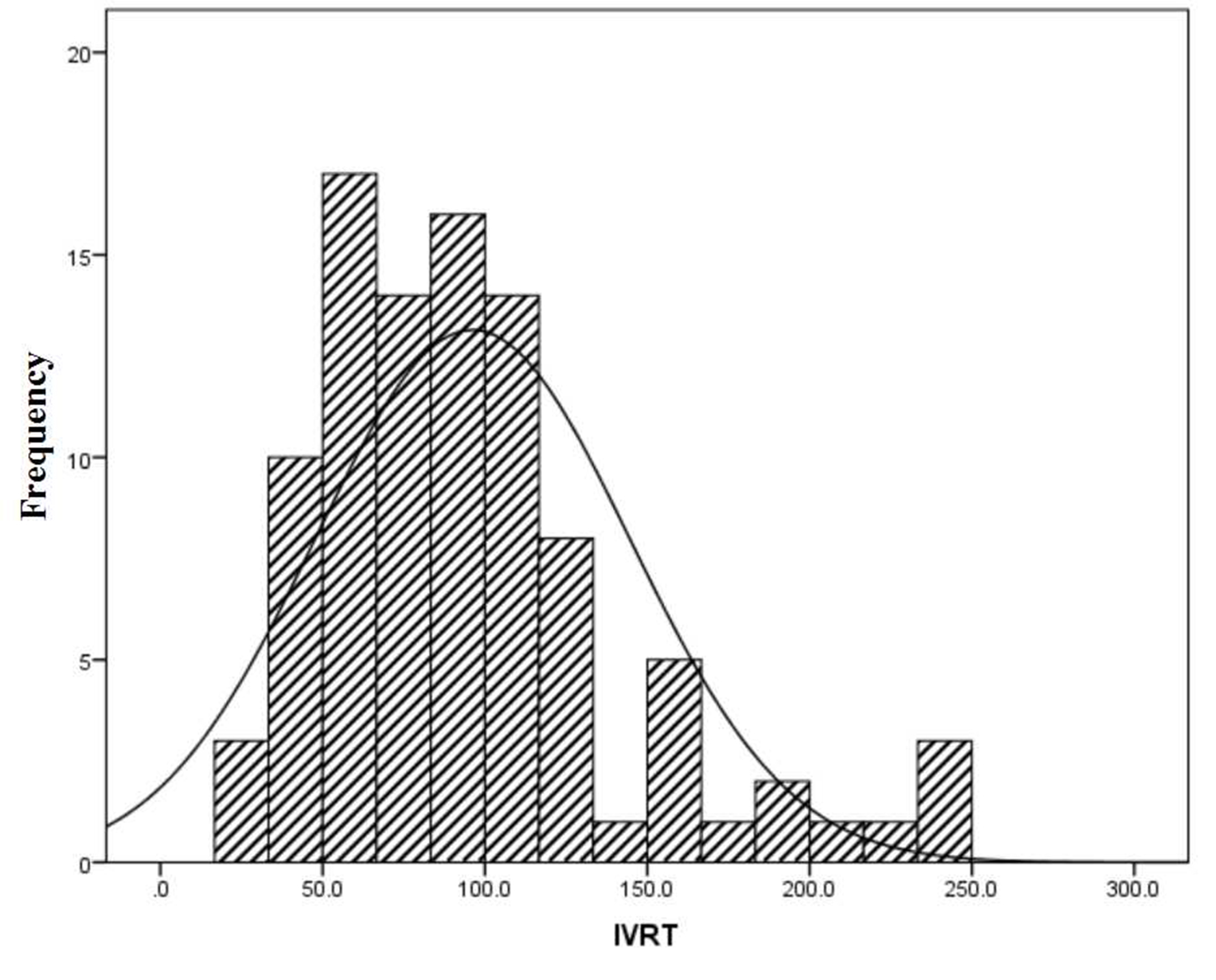

Supplement: Supplementary file 3 [file Image_3.JPEG]

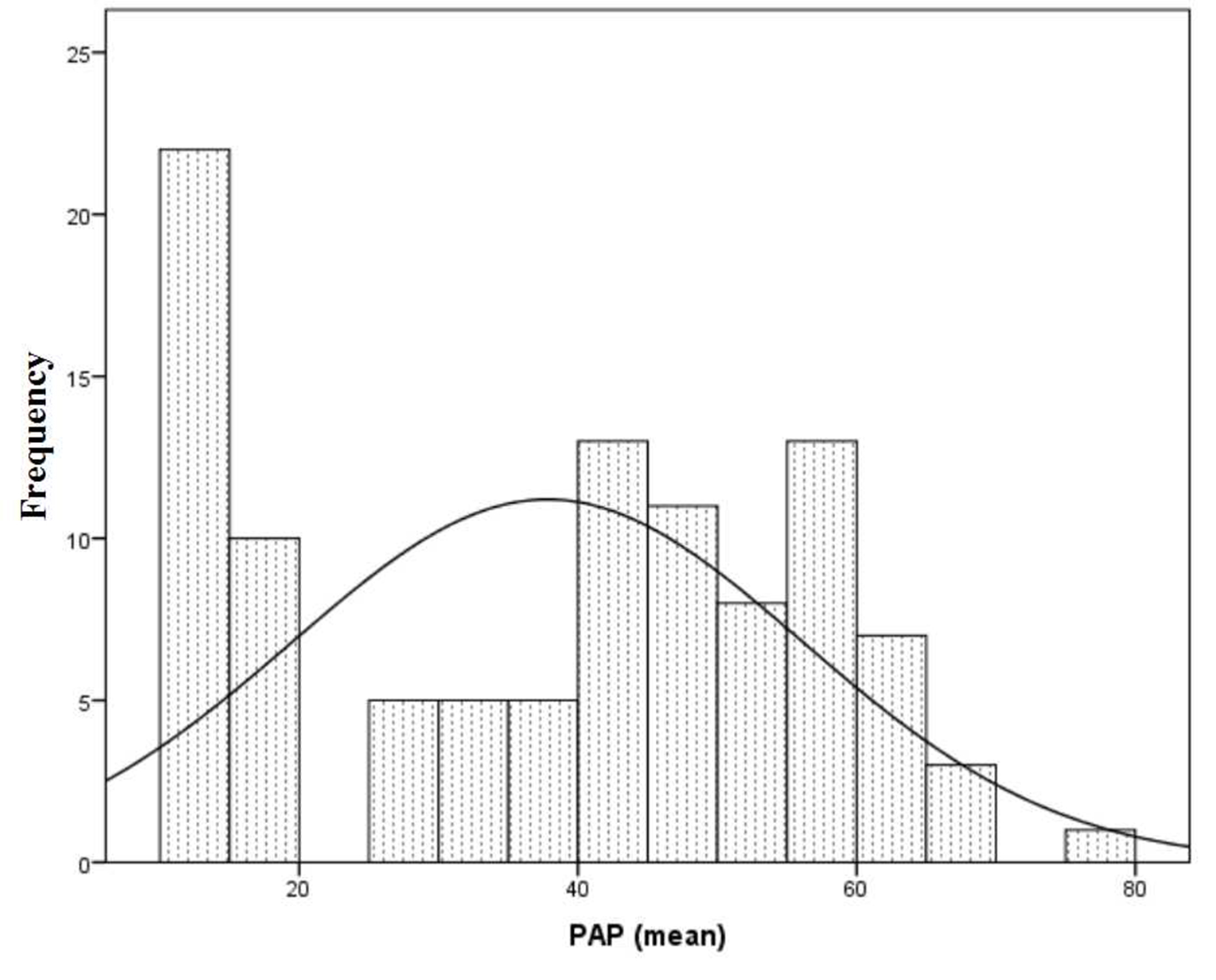

Supplement: Supplementary file 4 [file Image_4.JPEG]

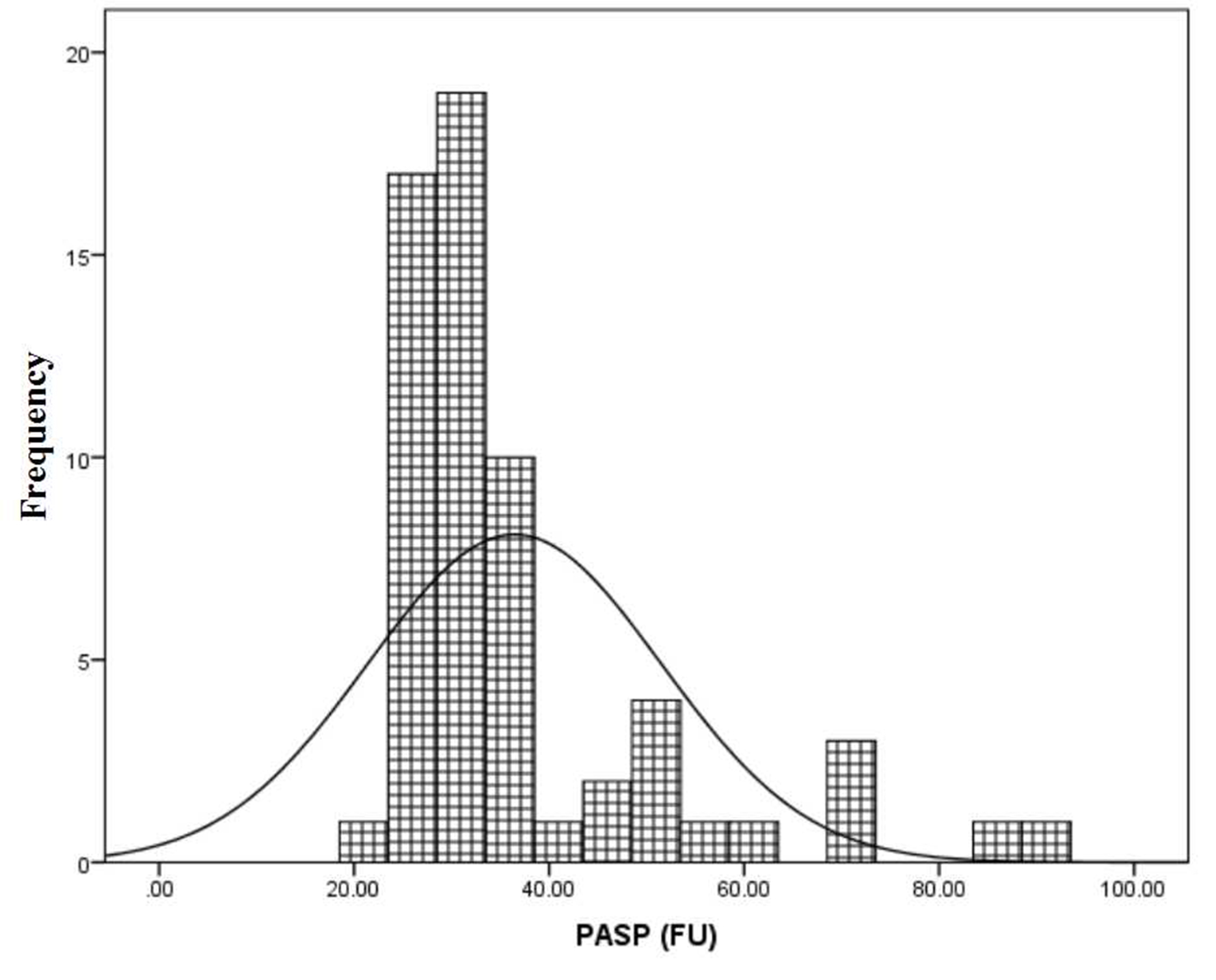

Supplement: Supplementary file 5 [file Image_5.JPEG]

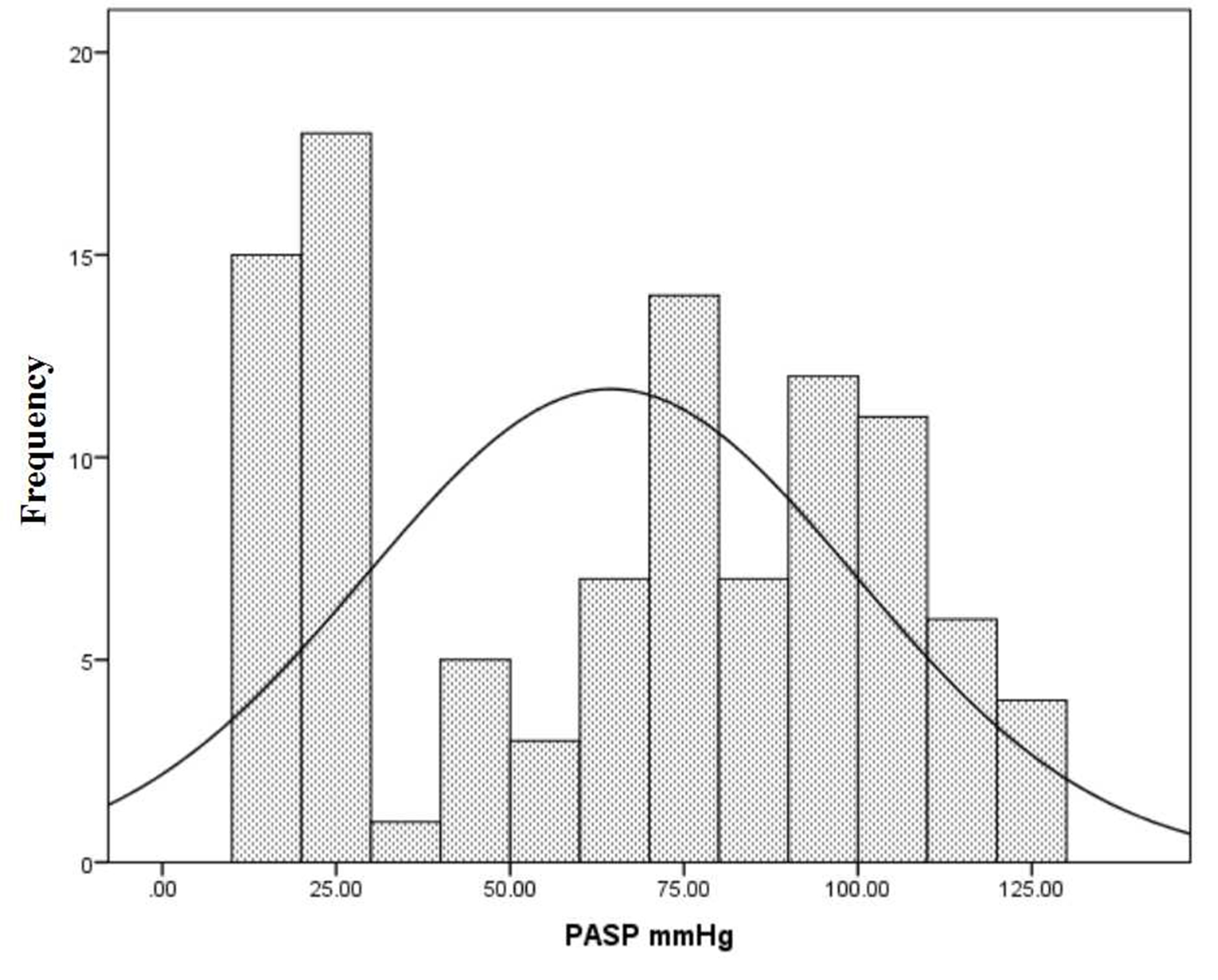

Supplement: Supplementary file 6 [file Image_6.JPEG]

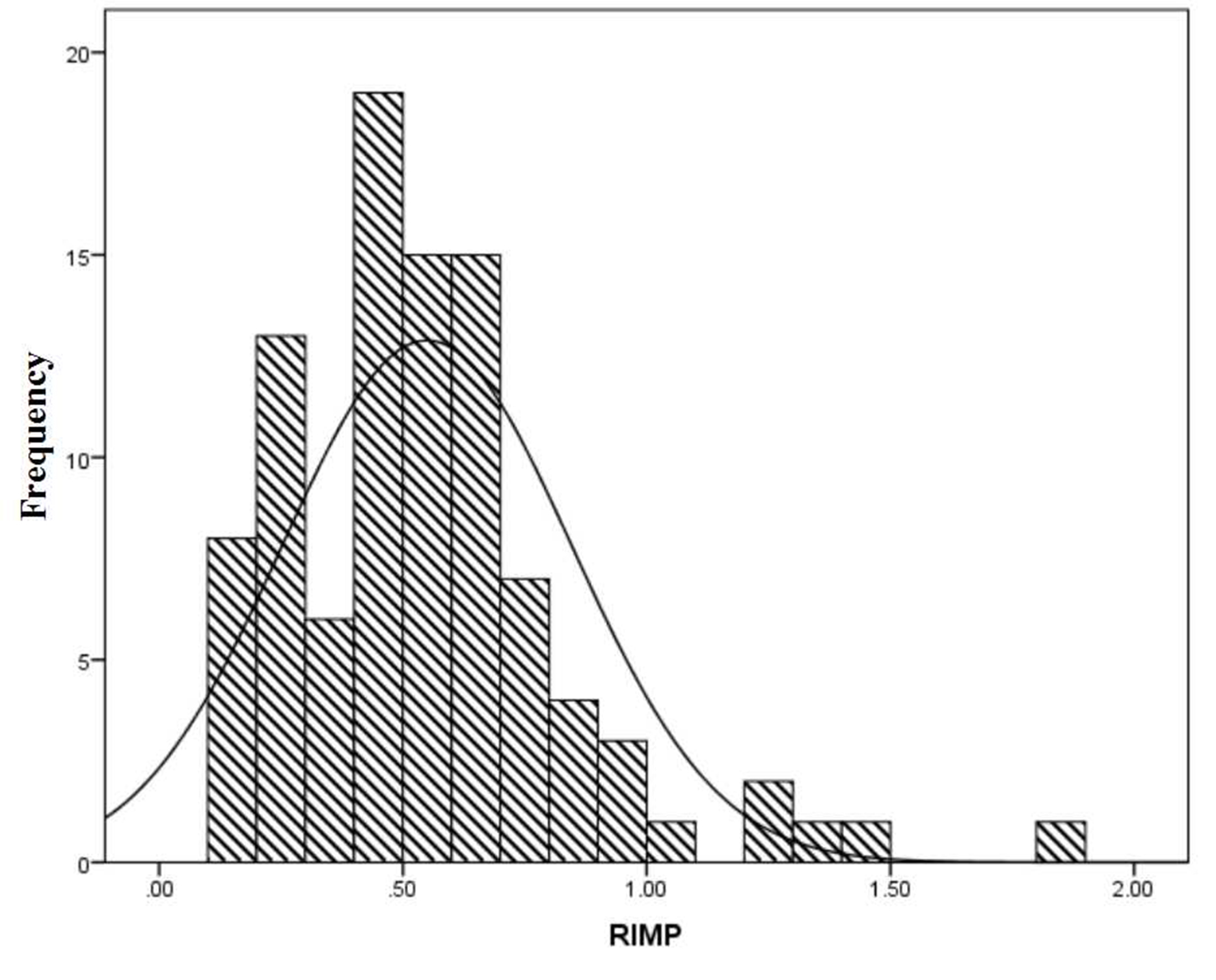

Supplement: Supplementary file 7 [file Image_7.JPEG]

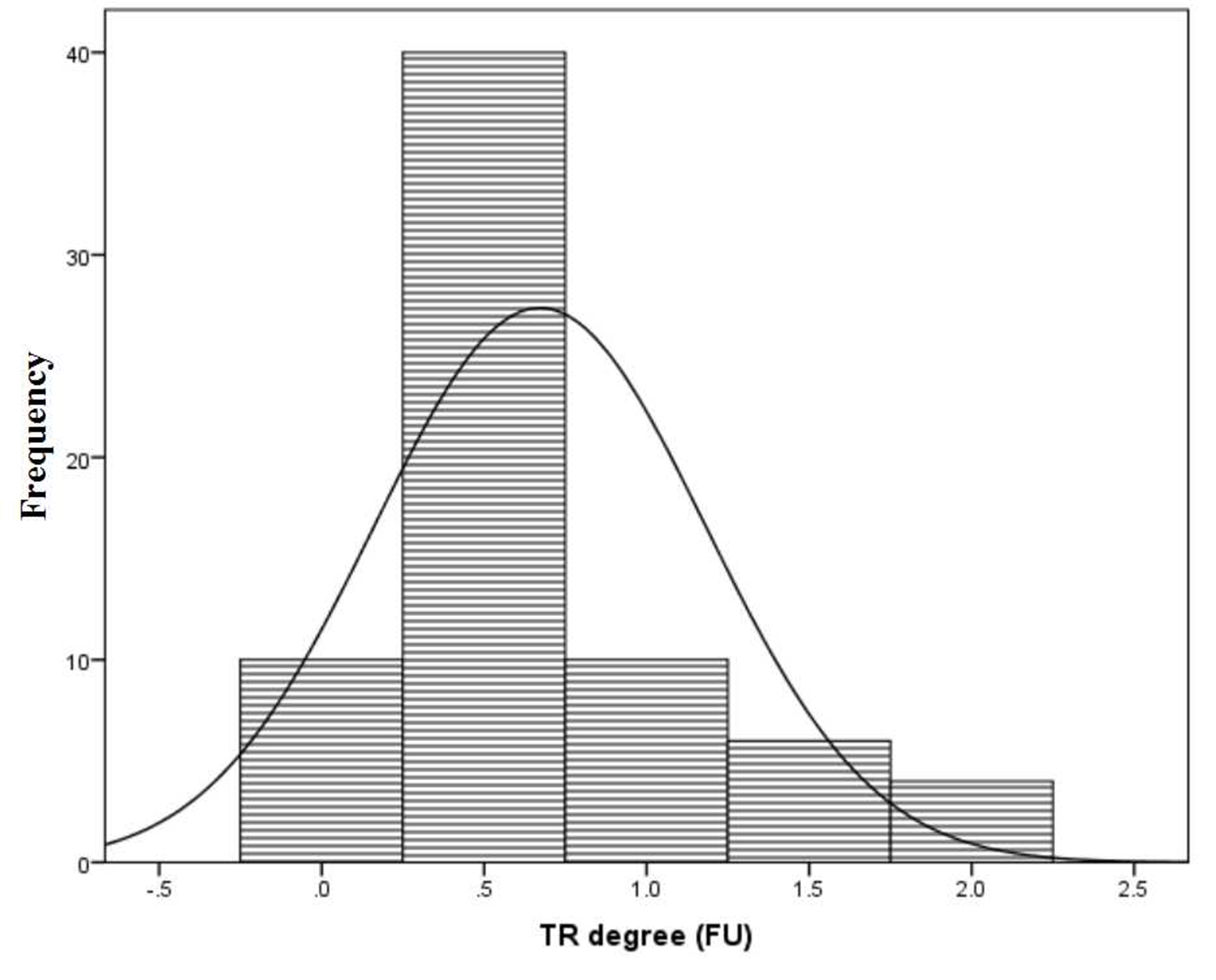

Supplement: Supplementary file 8 [file Image_8.JPEG]

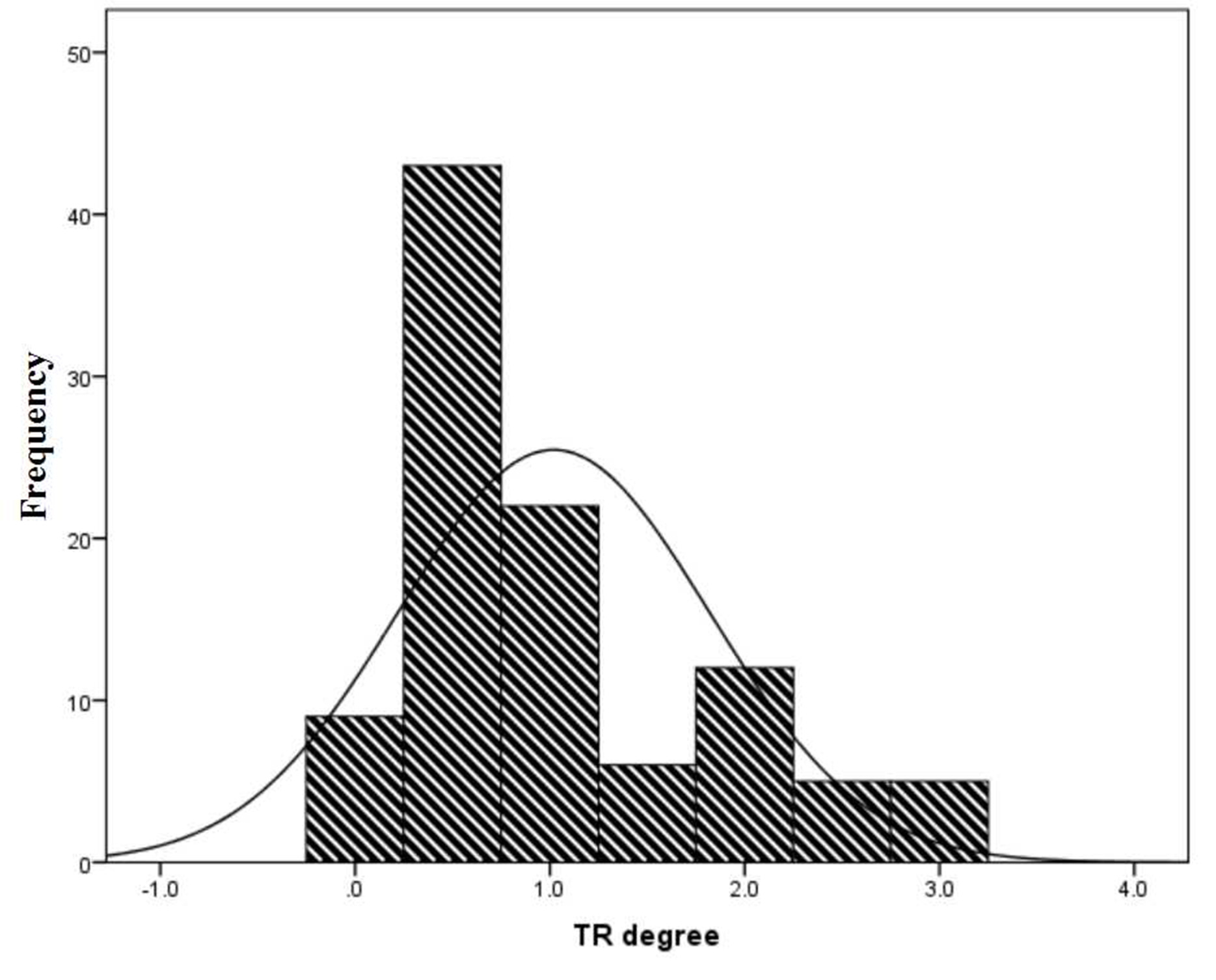

Supplement: Supplementary file 9 [file Image_9.JPEG]
